# Obesity and *STING1* genotype associate with 23-valent pneumococcal vaccination efficacy

**DOI:** 10.1172/jci.insight.136141

**Published:** 2020-05-07

**Authors:** Mathew Sebastian, Chu J. Hsiao, Hunter S. Futch, Robert S. Eisinger, Leanne Dumeny, Seema Patel, Mesfin Gobena, Divya S. Katikaneni, Joel Cohen, Anne-Marie Carpenter, Lisa Spiryda, Coy D. Heldermon, Lei Jin, Mark L. Brantly

**Affiliations:** 1 MD-PhD Training Program and; 2 Lillian S. Wells Department of Neurosurgery, University of Florida College of Medicine, Gainesville, Florida, USA.; 3 Genetics Institute, University of Florida, Gainesville, Florida, USA.; 4 Department of Anthropology, University of Florida College of Liberal Arts and Sciences, Gainesville, Florida, USA.; 5 Norman Fixel Institute for Neurological Diseases, Department of Neuroscience,; 6 Department of Pharmacotherapy and Translational Research and Center for Pharmacogenomics and Precision Medicine,; 7 Division of Pulmonary, Critical Care and Sleep Medicine, Department of Medicine,; 8 Department of Surgery, and; 9 Department of Obstetrics and Gynecology, University of Florida College of Medicine, Gainesville, Florida, USA.; 10 Division of Hematology and Oncology, Department of Medicine, University of Florida, Gainesville, Florida, USA.

**Keywords:** Infectious disease, Vaccines, Bacterial vaccines, Genetic variation, Obesity

## Abstract

**BACKGROUND:**

Obesity has been associated with attenuated vaccine responses and an increased risk of contracting pneumococcal pneumonia, but no study to our knowledge has assessed the impact of obesity and genetics on 23-valent pneumococcal vaccine (PPSV23) efficacy. We assessed the relationship of obesity (primary analysis) and stimulator of interferon genes (*STING1*) genotype (secondary analysis) on PPSV23 efficacy.

**METHODS:**

Nonobese (BMI 22–25 kg/m^2^) and obese participants (BMI ≥30 kg/m^2^) were given a single dose of PPSV23. Blood was drawn immediately prior to and 4–6 weeks after vaccination. Serum samples were used to assess PPSV23-specific antibodies. *STING1* genotypes were identified using PCR on DNA extracted from peripheral blood samples.

**RESULTS:**

Forty-six participants were categorized as nonobese (*n* = 23; 56.5% women; mean BMI 23.3 kg/m^2^) or obese (*n* = 23; 65.2% women; mean BMI 36.3 kg/m^2^). Obese participants had an elevated fold change in vaccine-specific responses compared with nonobese participants (*P* < 0.0001). The WT *STING1* group (*R232/R232*) had a significantly higher PPSV23 response than individuals with a single copy of *HAQ-STING1* regardless of BMI (*P* = 0.0025). When WT was assessed alone, obese participants had a higher fold serotype-specific response compared with nonobese participants (*P* < 0.0001), but no difference was observed between obese and nonobese individuals with 1 *HAQ* allele (*P* = 0.693).

**CONCLUSIONS:**

These observations demonstrate a positive association between obesity and PPSV23 efficacy specifically in participants with the WT *STING1* genotype.

**TRIAL REGISTRATION:**

ClinicalTrials.gov NCT02471014.

**FUNDING:**

This research was supported by the NIH and the University of Florida MD-PhD Training Program.

## Abstract 2

Obesity and a WT STING1 genotype are positively associated with efficacy of the 23-valent pneumococcal vaccine in a small cohort of subjects.

## Graphical abstract

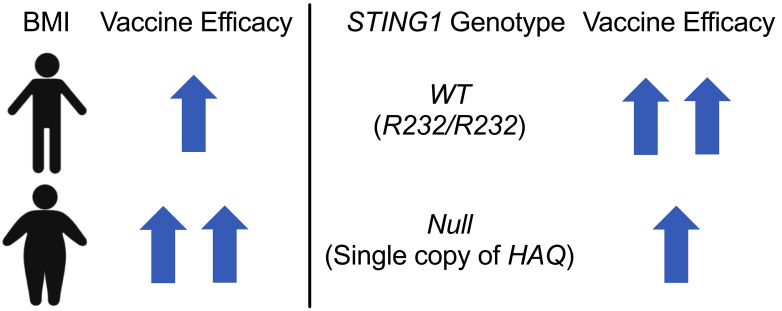

## Introduction

Pneumococcal infections are a leading cause of adult and childhood hospitalizations and mortality, with an estimated 1.6 million annual deaths (1, 2). A high BMI is associated with an increased risk for contracting pneumococcal pneumonia, suggesting that it is desirable to provide effective vaccination to this susceptible, and growing, population (3). A number of studies have demonstrated that obesity may be a risk factor for vaccine nonresponsiveness, including hepatitis B and tetanus vaccines, which are both protein based (4, 5). However, the effects of obesity on immune response to the polysaccharide-based pneumonia vaccine, which elicits a T cell–independent response, have not been characterized.

Obesity constitutes one of the largest public health issues in the world and is associated with widespread comorbidities, including insulin resistance, hypertension, cardiovascular disease, stroke, sleep apnea, cancer, chronic inflammation, and mortality (6–9). Though inflammation in obesity is multifaceted, recent attention has turned to genetic contributions of underlying inflammatory mechanisms (10, 11). In particular, studies have shown that obesity induces cytosolic mitochondrial DNA release, which triggers activation of STING (stimulator of interferon genes; refs. 12, 13). This obesity-induced activation of STING may impact vaccination response (14). Indeed, STING has been implicated in vaccine efficacy, as reduced antibody responses to T cell–independent type II antigens have been demonstrated in Sting-deficient mice (15). Also, Sting^–/–^ mice have impaired antibody responses to the 23-valent pneumococcal vaccine (PPSV23; Pneumovax 23), which also elicits a T cell–independent type II response (16). In humans, the *STING1* gene is highly heterogeneous, and R71H-G230A-R293Q (*HAQ*), the second most common human *STING1* allele, is carried by approximately 22.6% of Americans and approximately 63% of East Asians. A knockin mouse with the mouse equivalent of the human *HAQ-STING1* allele has an attenuated PPSV23 response (16, 17). Whether the *HAQ* allele influences PPSV23 response in humans is an unknown.

The aim of the Result of Obesity on Vaccine Efficacy (ROVE) Study was to utilize a nonrandomized controlled trial to evaluate, in otherwise healthy adults, the effect of obesity (BMI ≥30) versus nonobesity (22 ≤ BMI ≤ 25) on humoral responses to PPSV23 in relation to *STING1* genotypes. This is the first human study to our knowledge assessing the role of obesity and *STING1* genotype in PPSV23 efficacy. Stated as null, we hypothesized that levels of antibodies to serotypes against PPSV23 before and after vaccination in nonobese and obese participants would be identical.

## Results

### Participants

**Figure 1:**
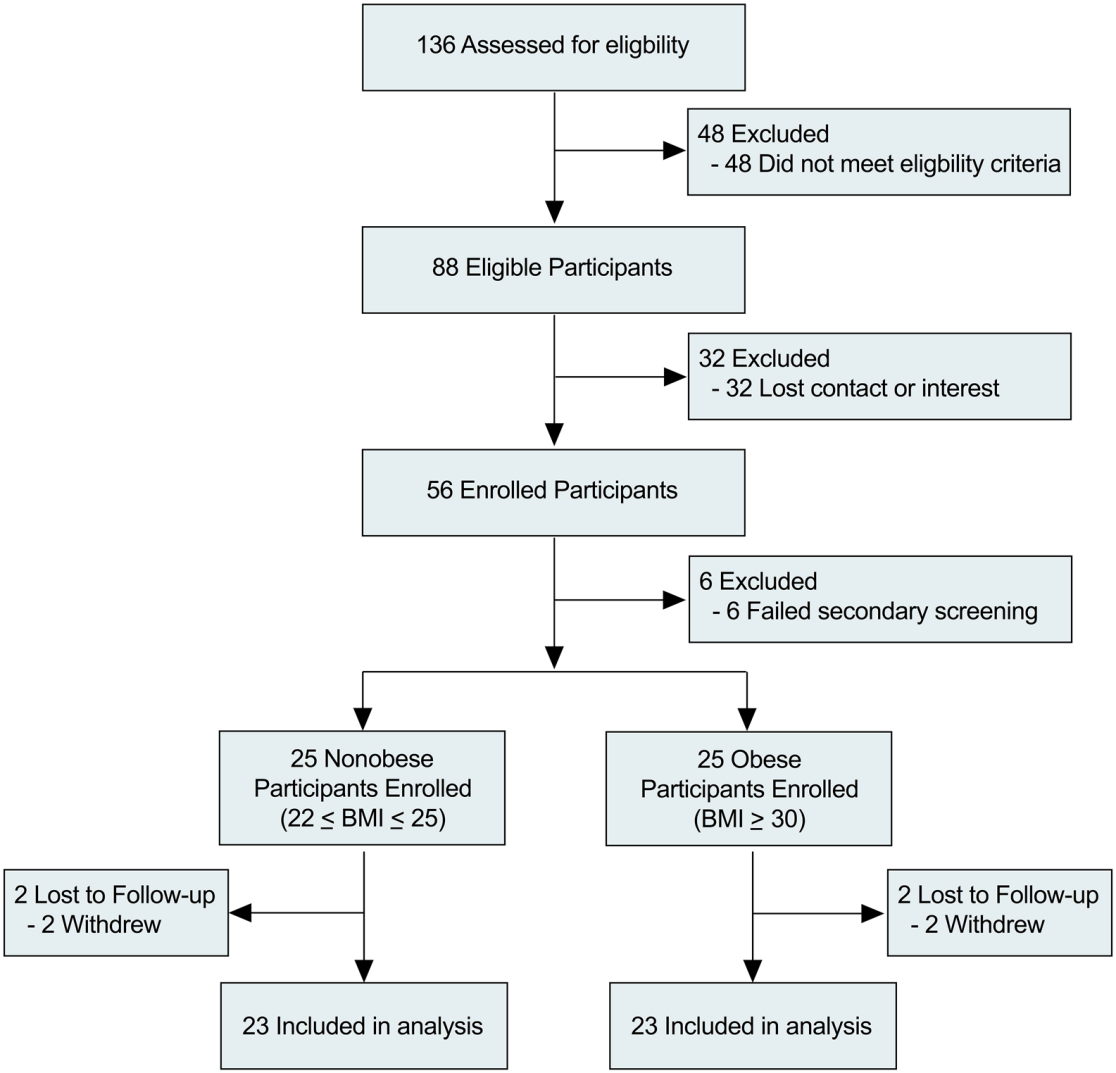
Flow diagram of participating subjects.

Enrollment began in January 2017 and was completed in August 2018 after the prespecified number of 23 subjects per group was reached. A total of 136 potential participants were interested in the study and invited to take a brief online survey to assess eligibility. Eighty-eight participants were eligible and, after 32 lost interest or were unable to be contacted again, 56 consented. Six participants failed secondary screening during the first visit. Of the 50 remaining participants, 25 had BMI between 22 and 25, and 25 had BMI >30. In each group, 2 participants withdrew from the study. In total, 23 nonobese and 23 obese participants completed both visits (Figure 1). The baseline characteristics of the 2 groups are shown in Table 1. The nonobese individuals were younger than the obese individuals (mean ± SEM, 95% CI: 23.0 ± 0.643, 21.7–24.4 vs. 29.0 ± 1.11, 26.7–31.3; *P* < 0.001). Participants in the obese group showed significantly elevated BMI, waist/hip ratio, WBC counts, hemoglobin A1C, erythrocyte sedimentation rate, and blood pressure compared with the nonobese group, which aligns with other studies involving obese participants (Table 1) (18–20). One adverse event occurred due to local swelling at the site of the vaccine injection, which resolved within 2 weeks.

### Outcomes

#### No difference in baseline serotype titers or seroconversion.

**Figure 2:**
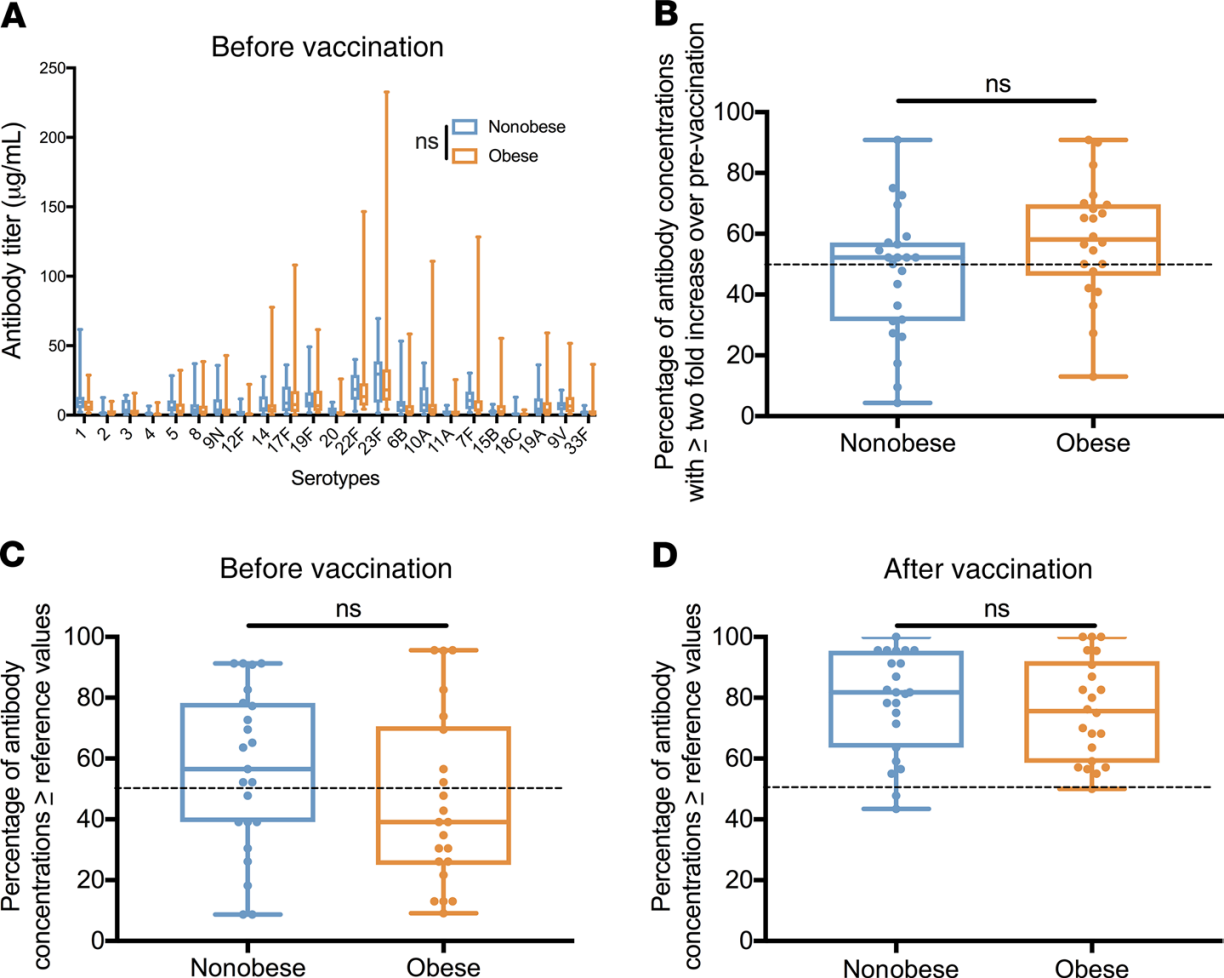
No difference in baseline PPSV23 titers or seroconversion. (**A**) Mean prevaccination titers to all serotypes among nonobese (*n* = 23) versus obese (*n* = 23) participants. (2-way ANOVA, *P* = 0.172). (**B**) Percentage of the 23 tested antibody concentrations per participant that were greater than or equal to a 2-fold increase over prevaccination levels in nonobese (*n* = 23) and obese (*n* = 23) participants (*t* test, *P* = 0.064). (**C**) Percentage of the 23 tested antibody concentrations that were greater than the reference values before vaccination in nonobese (*n* = 23) and obese (*n* = 23) participants (*t* test, *P* = 0.214). (**D**) Percentage of the 23 tested antibody concentrations that were greater than the reference values after vaccination in nonobese (*n* = 23) and obese (*n* = 23) participants (*t* test, *P* = 0.619). Segment inside the box indicates median; bounds of box represent 25th and 75th percentiles; and whiskers, minimum and maximum values.

Serum samples were collected both before and after vaccination (4–6 weeks later), and assessed for serotype-specific IgG responses. Since obesity is considered a risk factor for pneumonia, we measured prevaccination levels in nonobese and obese individuals, and found no significant difference (mean ± SEM, 95% CI: 8.06 ± 0.45, 7.18–8.94 vs. 9.3 ± 0.91, 7.51–11.1; *P* = 0.17; Figure 2A). Next, we evaluated whether there were clinically relevant differences in seroconversion between the groups. Participants in both groups showed equal clinical seroconversion that was normal for PPSV23 when either the 2-fold (Figure 2B) or the reference value (Figure 2, C and D) definition was used (see Methods). Overall, these data suggest there was no observable difference in the baseline status of serotype titers or in the rate of seroconversion between the nonobese and obese groups.

#### Obesity is positively associated with PPSV23 responses.

**Figure 3:**
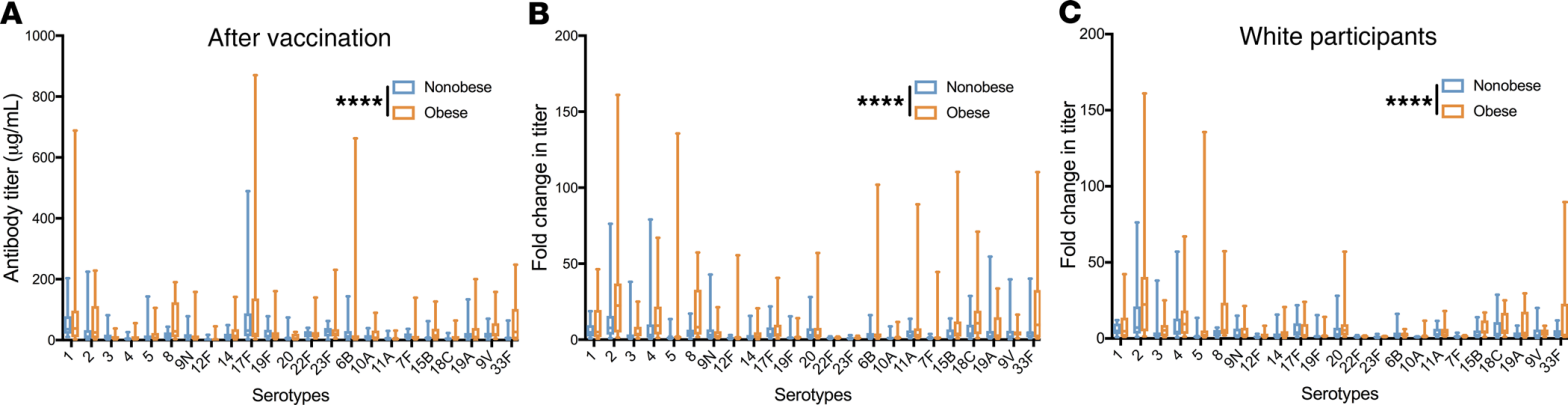
Obese participants have increased response to PPSV23. (**A**) Mean postvaccination antibody concentrations to all serotypes comparing nonobese (*n* = 23) and obese participants (*n* = 23) (2-way ANOVA). (**B**) Mean fold change to all serotypes comparing nonobese (*n* = 23) and obese participants (*n* = 23) (2-way ANCOVA). (**C**) Mean fold change to all serotypes among White participants only, comparing nonobese (*n* = 17) and obese participants (*n* = 12) (2-way ANOVA). Segment inside the box indicates median; bounds of box represent 25th and 75th percentiles; and whiskers the minimum and maximum values. ****P < 0.0001.

After vaccination, the overall concentration of anti-PPSV23 titers in the obese group were significantly elevated compared with the nonobese group (mean ± SEM, 95% CI: 34.1 ± 3.51, 27.2–40.9 vs. 19.8 ± 1.52, 16.8–22.8, *P* < 0.0001; Figure 3A). We next considered our primary end points. Controlling for age and sex, an ANCOVA showed a significant increase in fold change (mean ± SEM, 95% CI 9.56 ± 0.912, 7.77–11.4 vs. 4.75 ± 0.393, 3.98–5.23, *P* < 0.0001; Figure 3B) and log fold change (*P* < 0.001) in titers among obese participants. Post hoc comparisons showed that the obese group had a higher mean fold change in 21 of 23 serotypes (Supplemental Figure 1A; supplemental material available online with this article; https://doi.org/10.1172/jci.insight.136141DS1). Due to differences in the proportion of ethnicities in the study, we also assessed fold change differences in White participants only. At baseline, nonobese White participants had higher serotype-specific titers (mean ± SEM, 95% CI: 7.95 ± 0.522, 6.93–8.98 vs. 5.44 ± 0.477, 4.50-6.38; *P* < 0.0001; Supplemental Figure 1B). Though obese and nonobese White participants had similar antibody serotype-specific titers after vaccination (mean ± SEM, 95% CI: 21.7 ± 3.04, 15.7–27.6 vs. 19.4 ± 1.84, 15.8–23.0; *P* = 0.33; Supplemental Figure 1C), obese White participants had a significantly greater fold change increase (mean ± SEM, 95% CI: 8.07 ± 1.07, 5.96–10.2 vs. 4.33 ± 0.393, 3.56–5.10; *P* < 0.0001; Figure 3C). Together, these results suggest that obese participants mounted elevated PPSV23 IgG-specific responses compared with the nonobese group.

A timeline of 4–6 weeks after vaccination captures the latter portion of the primary humoral immune response. Since PPSV23 is a polysaccharide vaccine, we assessed IgM responses to serotypes that demonstrated the greatest group differences in IgG responses. The obese group had a significantly greater fold change in the selected IgM serotype–specific titers (mean ± SEM, 95% CI: 2.50 ± 0.20, 2.11–2.90 vs. 1.79 ± 0.118, 1.55–2.02; *P* = 0.002; Supplemental Figure 2A). Of the 5 IgM serotypes tested, all were elevated in the obese compared with the nonobese group (Supplemental Figure 2B). These results suggest that obese participants also have an elevated IgM response to specific serotypes compared with the nonobese group.

#### STING1 R232/R232 genotype is positively associated with the PPSV23 responses.

**Figure 4:**
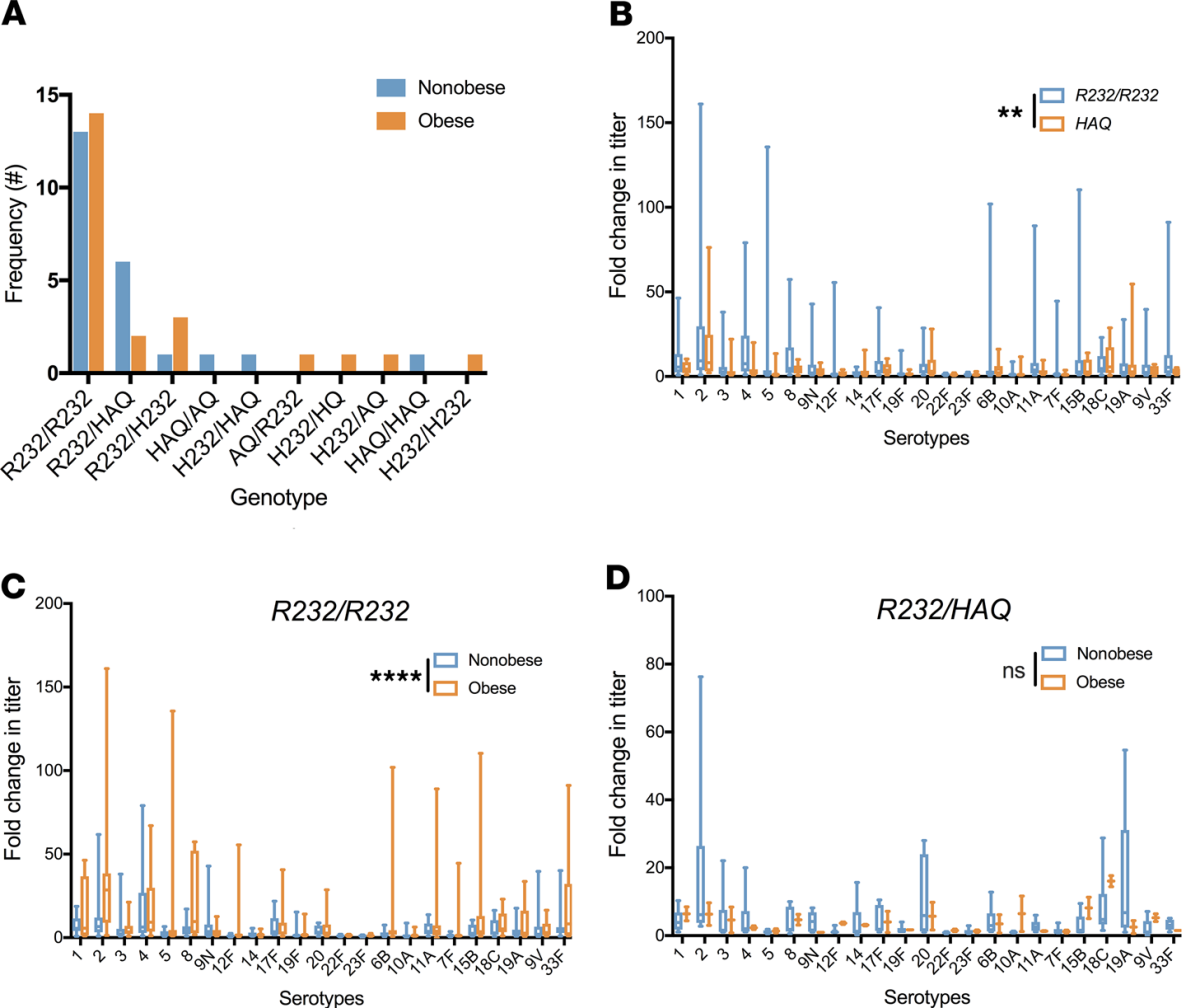
*R232/R232 STING1* genotype is positively associated with PPSV23 response. (**A**) *STING1* genotype frequencies in nonobese (*n* = 23) and obese populations (*n* = 23). (**B**) Mean fold change to all serotypes comparing *R232/R232* (*n* = 26) and any genotype with a copy of *HAQ* (*n* = 11) (2-way ANOVA, *P* = 0.0025). (**C**) Mean fold change to all serotypes among *R232/R232* participants only, comparing nonobese (*n* = 13) and obese participants (*n* = 14) (2-way ANOVA, *P* < 0.0001). (**D**) Mean fold change to all serotypes among *R232/HAQ* participants only, comparing nonobese (*n* = 6; 67% White) and obese (*n* = 2; 100% White) participants (2-way ANOVA, *P* = 0.693). Segment inside the box indicates median; bounds of boxes represent 25th and 75th percentiles; and whiskers, minimum and maximum values. **P < 0.01, ****P < 0.0001.

Next, we conducted genetic testing in our study population to assess the genotypes present for *STING1* in our secondary analysis. Ten genotypes were identified (Figure 4A). In the nonobese and obese groups, most participants had the WT genotype, *R232/R232* (13 of 23 and 14 of 23, respectively). Since the *HAQ* allele influences PPSV23 efficacy, specifically leading to an attenuated response in murine models, we assessed *HAQ* allelic frequency (16). Among the 11 participants who had the *HAQ* allele, 9 were in the nonobese group (Supplemental Figure 3). Next, we compared the 23 serotype-specific titers among the WT genotype, *R232/R232*, and any genotype that had a single copy of the *HAQ* allele. The WT genotype was associated with a significantly higher fold change in total anti-PPSV23 titers compared with any genotype that had a single copy of *HAQ* (mean ± SEM, 95% CI: 7.97 ± 0.74, 6.52–9.43 vs 4.46 ± 0.514, 3.45–5.48; *P* = 0.0025; Figure 4B). Thus, the *HAQ* allele in humans is associated with decreased fold change in anti-PPSV23 titers.

#### Obesity and the HAQ allele both affect PPSV23 response.

Next, we assessed the role of the *STING1* genotype in nonobese and obese participants. This analysis included only the genotypes *R232/R232* and *R232*/*HAQ*, as each was represented by at least 2 participants per nonobese or obese group. Among participants with the WT genotype, *R232/R232*, we found the obese group to have significantly higher anti-PPSV23 titers (mean ± SEM, 95% CI: 10.9 ± 1.37, 9.19–13.6 vs 5.09 ± 0.534, 4.04–6.14; *P* < 0.0001; Figure 4C). We then analyzed the limited number of participants with the *R232/HAQ* genotype who were nonobese (*n* = 6) and obese (*n* = 2), and found no difference (mean ± SEM, 95% CI: 4.61 ± 0.794, 3.04–6.18 vs. 4.14 ± 0.60, 2.93–5.35; *P* = 0.69; Figure 4D). For *R232/R232*, 20 of 23 serotype titers had higher means in the obese group (Supplemental Figure 4A), whereas for the *R232*/*HAQ* genotype, only 10 of 23 were higher in the obese group (Supplemental Figure 4B). Thus, obese participants with the *R232/R232* genotype had greater vaccine-specific responses, but this effect did not remain when obese individuals possessed 1 *HAQ* allele in this limited exploratory analysis.

## Discussion

As a growing population of obese individuals inevitably ages and becomes susceptible to pneumococcal infections, it is important to consider how obesity and genetics impact the efficacy of PPSV23. The ROVE study investigated the impact of obesity on immune response to the polysaccharide-based pneumococcal vaccination. Despite the fact that nonobese and obese participants had similar baseline serotype-specific titers and achieved similar clinically relevant responses, the obese group consistently had greater fold changes in serotype-specific titers compared with the nonobese group — the primary endpoint of ROVE. These data align with our observation that obese individuals had elevated inflammatory markers, such as erythrocyte sedimentation rate and WBC counts. Because studies regarding hepatitis B and tetanus vaccinations in obese participants have demonstrated reduced antibody response, our results were unexpected. This may be due to the nature of polysaccharide antigens involving the STING pathway. Indeed, Sting^–/–^ mice have decreased antibody production to PPSV23 (16).

Recently, the role of genetics in individual variability in weight has become better elucidated (21–23). Approximately 22.6% of Americans carry the *HAQ-STING1* allele (24). Intriguingly, we observed that 9 of the 11 individuals with a copy of *HAQ* were in the nonobese group. The WT genotype, *R232/R232*, was associated with significantly greater fold changes in total anti-PPSV23 titers compared with any genotype with a single copy of *HAQ*, regardless of BMI. When assessing only the WT genotype of *STING1*, *R232/R232*, we found that obese individuals had higher serotype-specific titers to the polysaccharide vaccine. However, this increase in titers was lost in obese individuals with a single copy of *HAQ*. Together, these exploratory data from our secondary analysis are the first to our knowledge to demonstrate that the *HAQ* allele is associated with a lower PPSV23 response in humans and are in line with published findings that the vaccine was ineffective in an *HAQ*-knockin mouse (16). Indeed, we found that the *HAQ* allele was enriched in the nonobese individuals. Future studies are needed to determine whether the *HAQ* allele is inversely associated with obesity.

Though our data suggest that the association of obesity and increased serotype-specific titers is related to a lack of *HAQ*, there may be other explanations. As PPSV23 is a polysaccharide vaccine, its efficacy depends on B cells. Immune cells play a critical role in the inflammatory state that accompanies obesity (25–27). Human adipose tissue in the obese state is known to secrete leptin and adiponectin (28, 29). Though macrophages and T cells have been implicated in the process of inflammation in obesity, emerging evidence suggests that B cells also modulate obesity-induced adipose tissue inflammation (30). Adipokines may alter B cell function and, in the context of a nonconjugated vaccine, may change the secretion of serotype-specific antibodies, as observed in this study.

Although overweight and obese individuals are at a significantly increased risk of pneumonia, a meta-analysis of 10 cohort studies on pneumonia-related mortality showed an inverse relationship between overweight and obese subjects and pneumonia-associated mortality (31). This “obesity survival paradox” could reflect our finding that obese individuals are more responsive to vaccination. It is thought that PPSV23 protects hosts from pneumococcal infection exclusively via production of opsonizing antibodies to capsular polysaccharides, with increases in serotype-specific antibody levels above reference thresholds leading to better clinical responses (32).

Among this study’s strengths is the finding that PPSV23 induces equal seroconversion in nonobese and obese individuals, suggesting that it is an effective vaccine in the obese population. The use of healthy volunteers in this study minimized some confounding variables and isolated obesity-mediated functions. This study provides findings that warrant future preclinical studies to assess whether enhanced vaccine-specific titers in obese individuals who have the WT *STING1* genotype can be extended to the 13-valent protein conjugate pneumococcal vaccine or other T cell–independent polysaccharide vaccinations, such as those against *Neisseria meningitidis* and *Salmonella enterica* serovar Typhi.

### Limitations.

Our study has several important limitations. First, like many human studies, our study cannot prove causality, in this case between obesity or *STING1* genotype and PPSV23 efficacy. Second, though the age range was set between 18 and 35 to avoid comorbidities due to age, a significant difference in age was observed between the 2 groups. Also, as adults between the ages of 18 and 35 are not generally indicated to receive PPSV23, our study may have limited application. The small cohort size and the even smaller pools of participants constituting subgroups (e.g., White individuals, *STING1* genotypes) limit the ability to rule out residual confounding effects and thus limit the external validity of the study. Last, a study that assessed the effect of obesity on influenza vaccine response found that though obesity correlated positively with the ability to mount a protective immune response at 1 month after vaccination, this increase was lost at 12 months after vaccination (33). However, this study used an inactivated vaccine that relies on a mechanism of action different from that of polysaccharide vaccines.

Overall, in this nonrandomized controlled trial, obese participants had greater serotype-specific antibody responses to PPSV23 than nonobese participants. When assessing the *STING1* genotype, we found that WT *STING1* obese participants had greater PPSV23 responses than nonobese WT *STING1* participants. The increases were lost when we assessed nonobese versus obese participants with the *HAQ-STING1* allele. In conclusion, our study indicated a stronger PPSV23 vaccine response in obese participants, which may lead to better antibody-mediated protection against pneumococcal infection.

## Methods

Further information can be found in the Supplemental Appendix, available online.

### Participants.

Recruitment for the study included the use of posted flyers, emails, and phone calls, and of recruitment databases such as University of Florida (UF) HealthStreet and ResearchMatch, a national health volunteer registry that was created by several academic institutions and supported by the NIH as part of the Clinical Translational Science Award program. Eligible participants were between 18 and 35 years of age, with either a BMI between 22 and 25 kg/m^2^; or ≥30 kg/m^2^ and a waist-to-hip ratio of at least 0.9 in males or 0.85 in females. Participants were excluded if they were pregnant, breastfeeding, diagnosed with diabetes, unable to fast for 8 hours prior to the initial blood draw, or previously immunized with PPSV23 or the 13-valent conjugate pneumococcal vaccine. Exclusion criteria also included use of immunosuppressive drugs, chemotherapeutic agents, or steroids; or a history of any of the following: pneumonia, splenectomy or damage to spleen, lung disease, history of chronic inflammatory disease, or autoimmune diseases. Subjects were also excluded if they planned to lose weight within the time frame of the study, experienced excessive weight loss or gain within 2 months prior to the study, planned to undergo any weight loss procedures during the study period, or partook in excessive weight training. The trial protocol, statistical analysis, and complete inclusion and exclusion criteria are presented in the Supplemental Appendix.

### Study procedures.

After passing an initial screening questionnaire, eligible participants were invited for 2 visits at the UF Clinical Research Center. During the first visit, the informed consent form was reviewed and signed. A physical exam, history, urine pregnancy test, and hemoglobin A1C test (<6.5%) were conducted to reassess eligibility with study criteria via a second screening. If these criteria were met, study nurses acquired baseline measurements, collected blood, and administered a single dose of PPSV23 intramuscularly (Merck, M043987 and N032092). The participants returned 4–6 weeks after vaccination for 1 additional blood draw. In compensation for their time and participation, participants were given US$50 for each visit completed.

### Outcomes.

Fasting blood samples were taken during both visits and analyzed by the UF Clinical Research Center for a complete blood count, complete metabolic panel, erythrocyte sedimentation rate, and total Igs. Serotype-specific IgG antibody responses against the 23 pneumococcal serotypes (i.e., 1, 2, 3, 4, 5, 6B, 7F, 8, 9N, 9V, 10A, 11A, 12F, 14, 15B, 17F, 18C, 19A, 19F, 20, 22F, 23F, 33F) were analyzed by Mayo Clinic Laboratories (test ID: PN23) using microsphere photometry. If the test was unable to quantitate specific serotypes due to a nonlinear dilution response, the interpretation of pneumococcal antibody serology was based on the remaining serotypes, as per the recommendations for the test (34). Clinically relevant responses to S*treptococcus pneumoniae* vaccination were assessed using the following 2 definitions: (i) antibody concentrations ≥the reference value for at least 50% of serotypes present in either a pre- or postvaccination sample; (ii) antibody concentrations increased by 2-fold or greater for at least 50% of serotypes present when comparing post- and prevaccination results. Since PPSV23 is a polysaccharide vaccine, IgM responses to serotypes that demonstrated the greatest group differences in IgG responses were assessed using ELISAs. The following reagents were used: pneumococcal polysaccharide type 2 (PPS2) (ATCC 500-X), PPS4 (ATCC 18-X), PPS8 (ATCC 503-X), PPS18C (ATCC 285-X), PPS33F (ATCC 67-X), ELISA Diluent (eBioscience 00-4202-56), TMB Solution 1× (eBioscience 00-4201-56), Stop Solution (Invitrogen SS04), ELISA Coating Buffer Powder (eBioscience 00-0044-59), Tween 20 (Fisher Scientific BP337-100), glycine hydrochloride (Acros Organics 411011000), and 10× PBS (Fisher Scientific BP3991).

### STING1 genotyping.

For assessment of *STING1* alleles, peripheral blood samples were drawn in EDTA-coated vacutainers, and DNA was extracted from buffy coats using a DNeasy Blood & Tissue Kit (QIAGEN 69504). The following primers were used for amplification: exon 3-4-5 Fwd 5′-GTCTGTTTTGTAGATCGAGAAATGG-3′, exon 3-4-5 Rev 5′-AGAATGGTCATGGATTTCTTGG-3′; exon 6 Fwd 5′-CAGCTAGGGACACTACAGCTCAGA-3′, exon 6 Rev 5′-CTGGCCTCCTGTACAATGAGAGT-3′; exon 7a Fwd 5′-CTCCATAGCCCCTTCTGACTCTT-3′, exon 7a Rev 5′-GGCTTAGTCTGGTCTTCCTCTTACC-3′. PCR products were purified using QIAquick PCR purification kit (QIAGEN 28104) and run on agarose gel for product size confirmation. Products were then sent to GENEWIZ for sequencing.

### Statistics.

The study was powered based on previous work assessing the effect of atorvastatin on immune response to PPSV23 (35). The sample size was calculated by comparing the antibody titer of each serotype to its specific reference value to determine whether a subject mounted a positive immune response per titer. Subjects received a score from 0 to 14 corresponding to the number of positive titers. The cutoff value used for sample size calculation was a BMI of 25. For the BMI <25 group, the mean number of positive titers was 9.2, with an SD of 2.3; and for the BMI >25 group, the mean was 7.4, with an SD of 1.5. At a significance level of α = 0.05 for a 2-sided *t* test at 80% power, 23 subjects per group was necessary.

Our primary outcome variable was fold change in titer levels from before to after vaccination. In our primary analysis, we used an ANCOVA to test for differences in fold change titer levels across groups (nonobese and obese) while controlling for sex and age. Our secondary outcome was analyzed using 2-way ANOVA to assess fold change in titer levels between the *STING1 WT* genotype, *R232/R232*, and genotypes that include the *HAQ* allele. Secondary analyses were interpreted as exploratory. In post hoc analyses, we examined titers for each of the specific antipneumococcal antibodies without multiple *P* value correction. For data comparisons between 2 populations, as seen in the baseline characteristics table, normality was assessed by Shapiro-Wilk test, followed by either an unpaired 2-sided *t* test for parametric data or Wilcoxon’s signed-rank test for nonparametric data to assess for differences between groups for individual serotype-specific titers, complete blood count, complete metabolic panel, erythrocyte sedimentation rate, and vaccine efficacy due to *STING1* genotype between groups. All hypothesis tests were 2 sided, and a *P* value less than 0.05 was considered significant. A χ^2^ test was used to determine differences in sex and ethnicity. Data were analyzed in Prism (GraphPad) and R 3.5 (http://r-project.org).

### Study approval.

This study was approved by the UF Institutional Review Board (IRB 201401069). Written informed consent was obtained from all participants.

## Author contributions

MS, RSE, LD, CJH, HSF, AMC, LJ, and MLB participated in the study design. MS, RSE, LD, CJH, HSF, AC, SP, MG, DSK, and JC participated in acquisition, analysis, and interpretation of data. MS wrote the initial draft of the manuscript, which was reviewed, edited, and approved by all authors. MS, RSE, LD, CJH, and HSF conducted primary data analysis. MS, RSE, LD, CJH, HSF, LJ, and MLB participated in administrative, technical, and material support. Study supervision was provided by LS, CDH, LJ, and MLB. Authorship was assigned among co–first authors in reverse alphabetical order.

## Supplementary Material

Supplemental data

## Figures and Tables

**Table 1:**
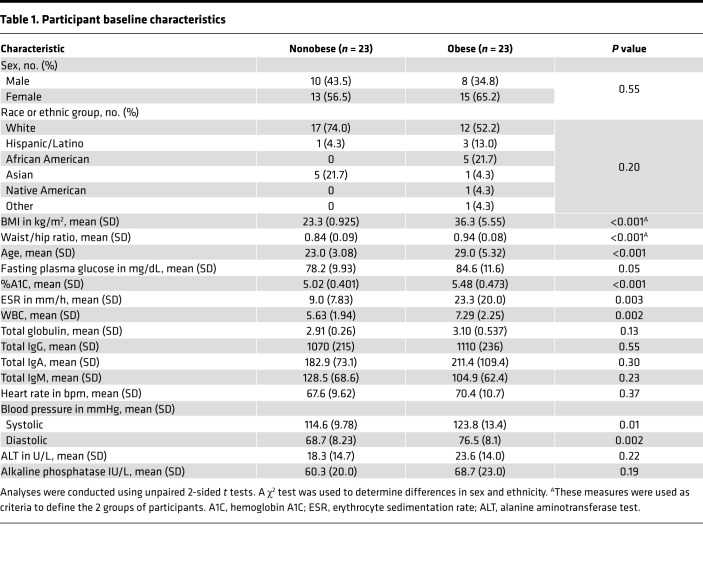
Participant baseline characteristics

## Version 1 05/07/2020

Electronic publication
