# Supplementary material for: Obesity and STING1 genotype associate with 23-valent pneumococcal vaccination efficacy
Source: JCI Insight. 2020 May 7;5(9):e136141. doi: 10.1172/jci.insight.136141 (PMC7253011; doi:10.1172/jci.insight.136141)
Supplement: Supplemental data [file jciinsight-5-136141-s006.pdf]

Supplemental Figure 1

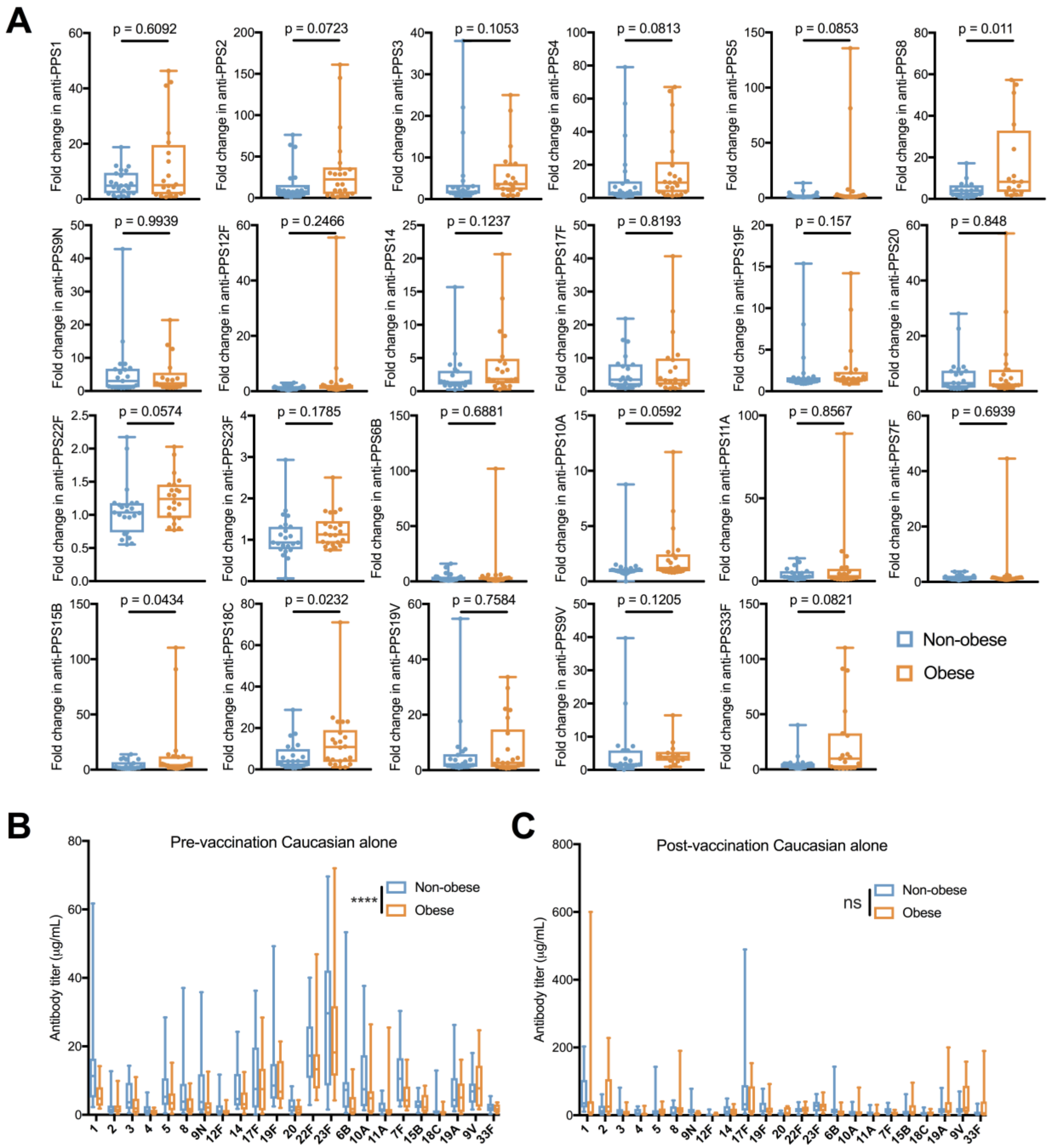

Individual fold-change comparisons and antibody titers of Caucasians pre- and post-vaccination. A) Individual serotype-specific IgG fold change comparisons of non-obese (orange) vs. obese (blue). Comparisons were made using a Mann-Whitney two tailed test. Unadjusted *P* values shown. B) Mean pre-vaccination antibody concentrations to all serotypes among Caucasians only comparing non-obese vs. obese participants (mean±SE, 95% CI 7.95±0.522, 6.93-8.98 vs. 5.44±0.477, 4.50-6.38, 2-way ANOVA, $P < 0.0001$). C) Mean post-vaccination antibody concentrations to all serotypes among Caucasians only comparing non-obese vs. obese participants (mean±SE, 95% CI

19.4±1.84, 15.8-23.0 vs. 21.7±3.04, 15.7-27.6, 2-way ANOVA, $P = 0.333$). Segments inside the box indicates median, box edges represent 25th and 75th percentiles and whiskers the minimum and maximum values. ns = not significant, ****$P < 0.0001$

Supplemental Figure 2

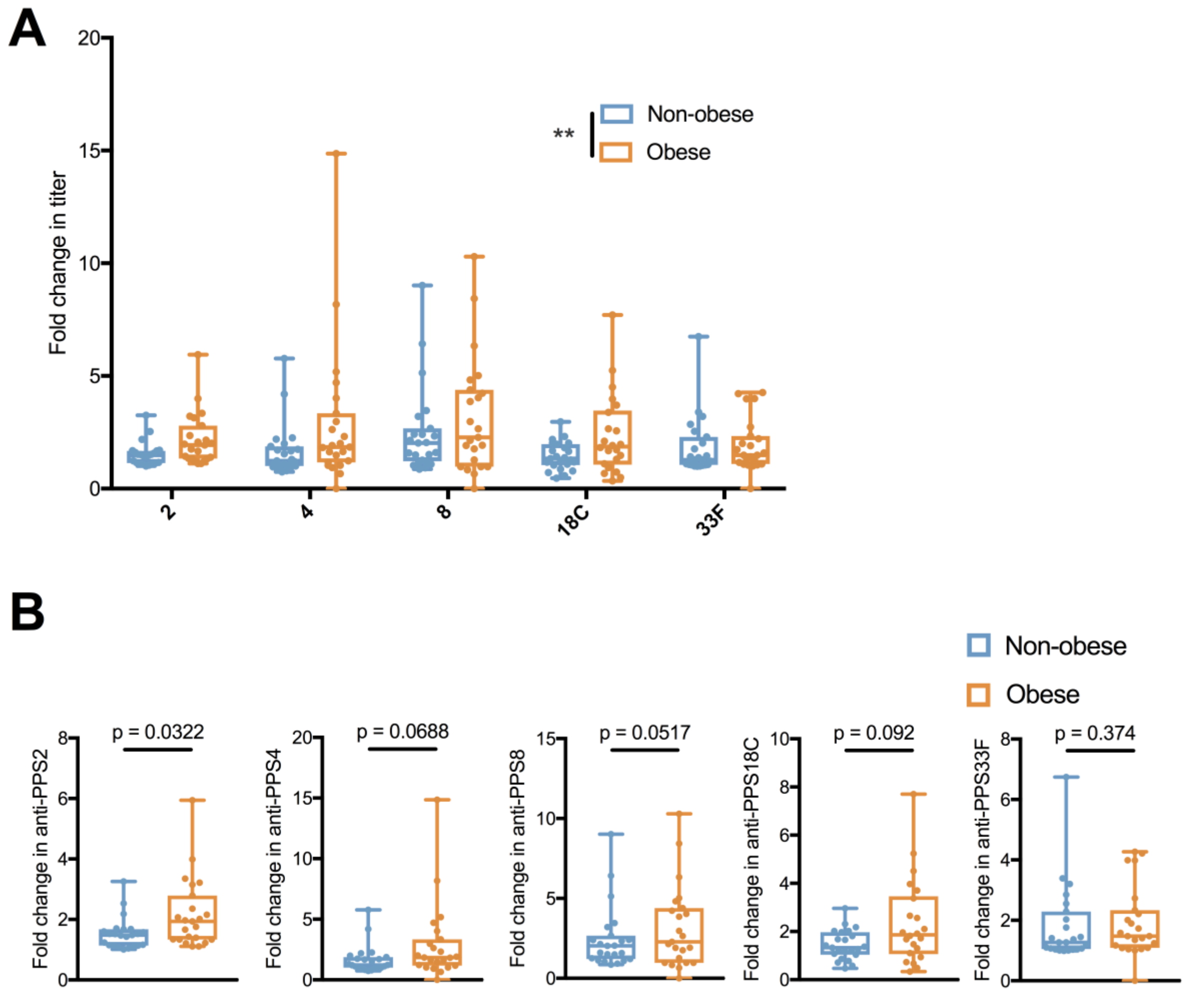

PPSV23-specific IgM fold change responses A) Comparison of non-obese and obese IgM titers (mean±SE, 95% CI 1.79±0.118, 1.55-2.02 vs. 2.50±0.20, 2.11-2.9, 2-way ANOVA, $P = 0.002$). B) Individual comparisons of non-obese and obese IgM titers were made using a Mann-Whitney two tailed test. Unadjusted p values shown. Segments inside the box indicates median, box edges represent 25th and 75th percentiles and whiskers the minimum and maximum values. $^{**}P < 0.01$

Supplemental Figure 3

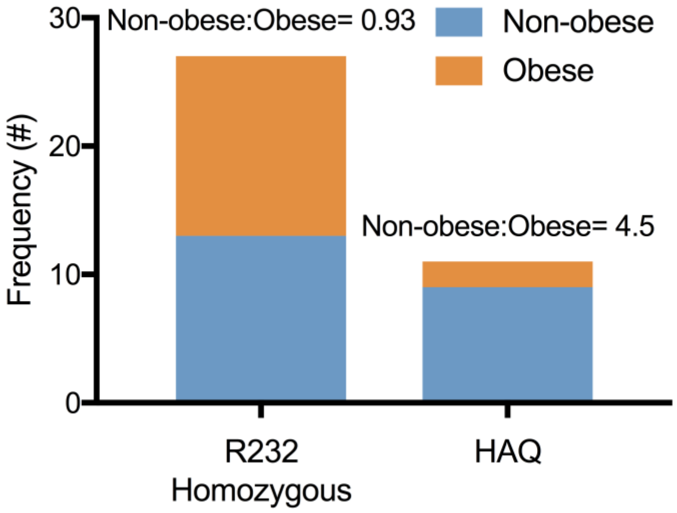

Proportion of non-obese and obese participants with *STING1* genotypes *R232/R232* and any genotype with one copy of *HAQ*.

Supplemental Figure 4

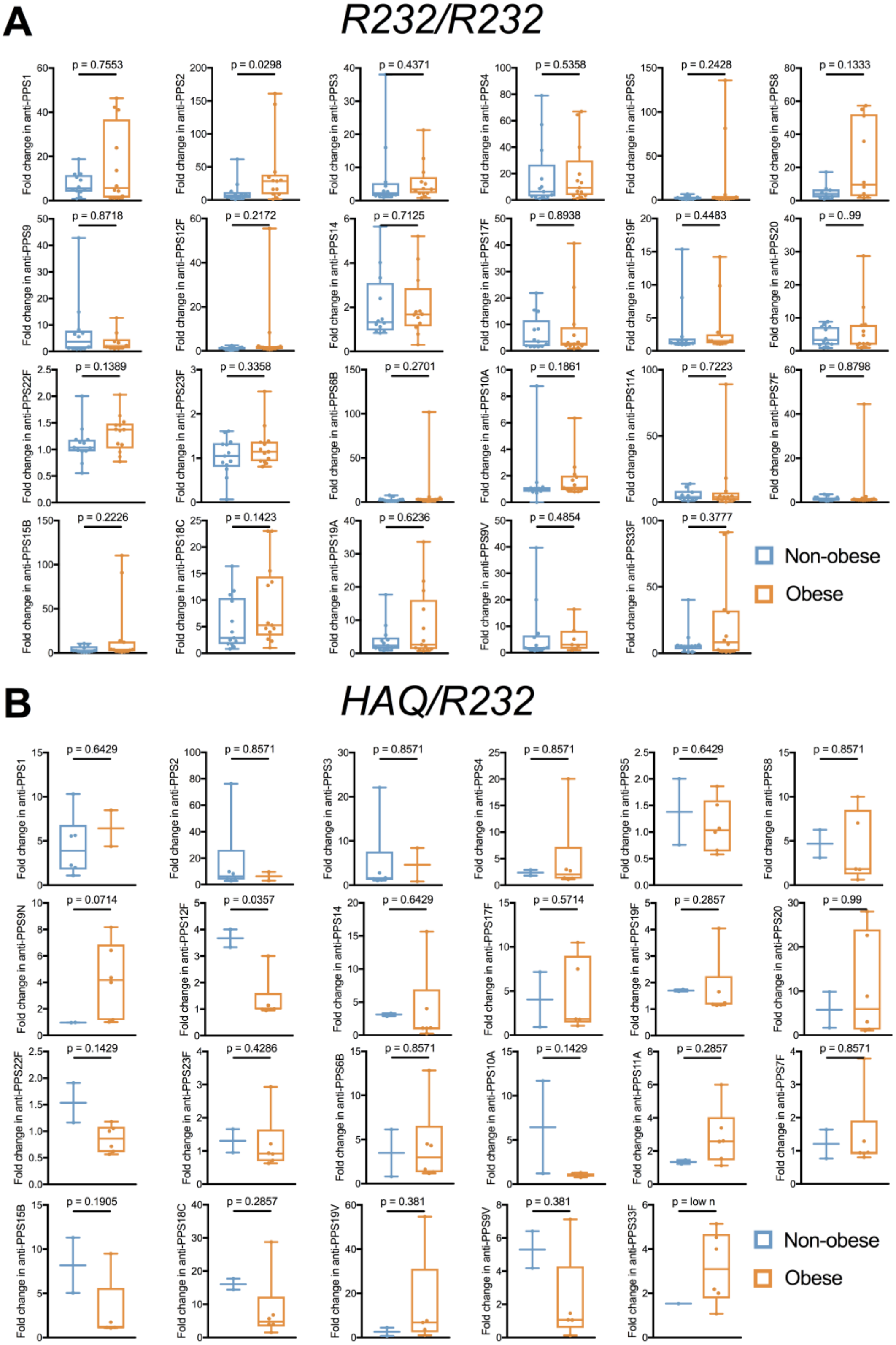

Individual fold-change comparisons for *R232/R232* or *HAQ/R232.* Individual serotype-specific IgG fold change comparisons of non-obese (orange) vs. obese (blue) among A) *R232/R232* or B) *R232/HAQ* genotypes. Comparisons were made using a Mann-Whitney two tailed test. Unadjusted *P* values shown. Segments inside the box indicates median, box edges represent 25th and 75th percentiles and whiskers the minimum and maximum values.

Supplemental Appendix

CTSI Protocol Number	
Project Title	The Effect of Obesity on Immune Responses to Pneumococcal Polysaccharide Vaccine<br><br>Short Title: Pneumonia Vaccine Study
Funding Source(s)	CTSI, MD-PhD Program
Grant Number(s)	
IRB Number	IRB201401069
IRB Expiration Date	
Project PI	Mark Brantly, M.D.
PI's Department	Medicine
PI's Phone Number/Email,	(352) 392-2666 / brantml@medicine.ufl.edu
Co-Investigator(s)	Leanne Dumeny, Robert Eisinger, Hunter Futch, Chu Jane Hsiao, Lei Jin Ph.D. Mathew Sebastian, Wei Wang,

CTSI Projected Utilization

Study Type: Pilot, Single Center

	2015	2016	2017	2018	2019
# Inpatient Days	0	0	0		
# Outpatient Visits	0	0	Visit 1: 86 participants<br><br>Visit 2: 56 participants		
# Scatter-Bed Hours	0	0	0		

CTSI Resources Required

Nursing Intensity	Medium
Bionutrition Intensity	None
Specimen Processing Laboratory	Yes
Biomedical Mass Spec Laboratory	No
DNA Bank	No
DNA Core Lab	No
Data Systems Lab Intensity	Medium
**CRC Staff will complete:**	
Ancillary Cost per Inpatient Day	$
Ancillary Cost Per Outpatient Visit	$

1. ABSTRACT:

Obesity (a body mass index ≥ 30 and waist to hip ratio of at least 0.9 in males and at least 0.85 in females) has been correlated with higher mortality risk and is associated with a proinflammatory state (e.g. elevated levels of TNF-alpha and interleukin-6). Obesity has also been associated with a lower immune response to various types of vaccination (e.g. influenza, hepatitis B, tetanus). As the prevalence of obesity increases, so does the need for a deeper mechanistic understanding of how obesity attenuates vaccination response. It is important to assess the effect of obesity on the immune response to vaccines. The pneumococcal polysaccharide vaccine (Pneumovax 23) is a safe method for stimulating an immune response and its effect on obesity has not been elucidated. As a growing population of obese individuals inevitably age and become susceptible to pneumococcal infections, it is important to consider how obesity may impact the efficacy of Pneumovax 23. We hypothesize that a chronically stressed immune system in obese individuals will be unable to respond as well to vaccination compared to individuals with a BMI between 22 and 25. We will utilize a prospective cohort design. We will also conduct various surveys to examine psychosocial factors that may influence the interaction between obesity and immune response to vaccination. We will also perform genotyping of STING to assess how it may influence the relationship between obesity and immune response to vaccination.

2. HYPOTHESIS AND SPECIFIC AIMS:

The primary objective of this study is to demonstrate the effect of obesity on the immune system's ability to mount a protective immune response against the Pneumococcal Polysaccharide Vaccine (Pneumovax 23).

Primary Hypothesis (stated as null): Levels of antibodies to serotypes in Pneumovax 23 in pre- and post- vaccination in individuals with a BMI between 22 and 25 and obese individuals are identical.

Secondary Hypothesis (stated as null): Levels of pro- and anti-inflammatory cytokine levels pre- and post-vaccination in individuals with a BMI between 22 and 25 and obese individuals are identical.

Tertiary Hypothesis (stated as null): The perceived level of stress, loneliness, social support, and dispositional optimism will be the same between the group of individuals with a BMI between 22 and 25 and the group of obese individuals, or between groups with different immune responses to the vaccine.

Quaternary Hypothesis (stated as null): Those who carry *STING* variants and those who are wildtype *STING* carriers will not have differing immune responses to Pneumovax 23.

Specific Aim 1: Quantify changes in the adaptive immune response to Pneumovax 23 by measuring specific antibodies to Pneumovax 23 antigens pre- and post-vaccination in the group of individuals with a BMI between 22 and 25 and the group of obese individuals.

Specific Aim 2: Quantify changes in the inflammatory immune response to Pneumovax 23 by measuring C-reactive protein (CRP) and cytokines pre- and post-vaccination in the group of individuals with a BMI between 22 and 25 and the group of obese individuals.

Specific Aim 3: Determine if perceived stress, loneliness, social support, and dispositional optimism are associated with adaptive immune response to Pneumovax 23.

Specific Aim 4: Determine how STING genotypes may affect the adaptive immune response to Pneumovax 23.

3. BACKGROUND AND SIGNIFICANCE:

Pneumococcal infections are a leading cause of adult and childhood hospitalization and mortality with an estimated 1.6 million deaths occurring annually.[1,2] Prophylactic measures against pneumococcal infections exist in the form of a vaccine called Pneumovax 23. While Pneumovax 23 is approved for use in all adults 50 years or older and individuals as young as 2 years who are at increased risk for infection, the vaccine is recommended for all adults 50 years and older or adults age 19 to 64 with conditions or treatments that affect the immune system.[3] These recommendations were developed, in part, from a 14-year cohort study comparing the proportion of pneumococcal infections caused by varying pneumococcal serotypes included in vaccinated and unvaccinated individuals.[4] Pneumovax 23 had an overall efficacy rate of 57% in preventing pneumococcal infection. In addition to recommending the vaccine to the elderly, the vaccine is also recommended for individuals who have been identified as being at higher risk for contracting pneumococcal pneumonia, such as those infected by human immunodeficiency virus.[5] Increased weight gain and a high body mass index have been associated with an increased risk for contracting pneumococcal pneumonia, suggesting it is desirable to provide effective vaccination of this susceptible population.[6]

Studies have shown that obesity is implicated in decreased immunological responses to hepatitis B, tetanus, and influenza vaccines.[8,9,10] For example, obesity is associated with a lower vaccine-specific IgG titer (<10mIU/mL) following hepatitis B vaccination.[11] Furthermore, obesity alters the metabolic and endocrine status of individuals that leads to immune dysfunction.[12] Current evidence suggests that a number of proinflammatory markers and tests are elevated in obesity such as TNF, IL-6, CRP, and erythrocyte sedimentation rate.[13, 14, 15] This pro-inflammatory state may attenuate the initial humoral immune response that vaccinations provide.[9] Additionally, obesity has been associated with a pro-inflammatory state (elevated levels of TNF-alpha and interleukin 6), impaired natural killer cell activity, decreased $CD8^+$ T cell populations, and decreased response to antigen stimulation, which may affect responses to vaccination (Figure 1).[16,17,18] Immunizations in obese patients may also be ineffective due to an inadequate needle length in depositing the vaccine intramuscularly through subcutaneous fat tissue.[18]

No current studies have examined the effects obesity may have on the response to pneumococcal vaccine in humans. We hope to initiate an observational prospective cohort study to investigate the effect of obesity on immune responses in individuals vaccinated with Pneumovax 23.

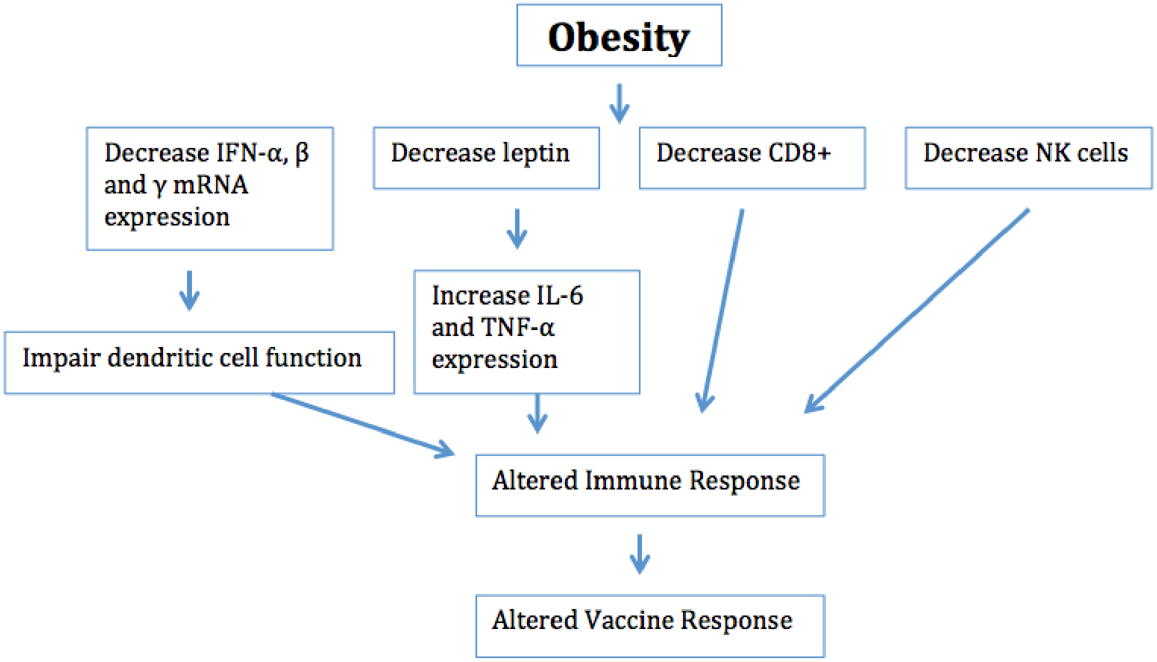

Figure 1. Mechanism of Obesity on Immune Response to Vaccination. Modified from Hur *et al.*, 2013.

In addition to the factors that alter the immune response in obesity, various psychosocial factors such as stress, loneliness, social support and dispositional optimism have been documented to influence the immune system. Studies have shown stress-related dysregulation of the immune system is large enough to impact health.[19,20] Specifically, stress has been shown to be associated with decreased immune response to vaccination such as less seroconversion, lower IgG antibody response, and down-regulation of the immune response.[21,22,23] Loneliness, too, has been shown to be associated with poorer antibody response to the influenza vaccine and impaired cellular immunity.[24,25] Greater social support appears to generate a stronger immune response to a vaccine[21] and is related to lower levels of interleukin-6.[26,27,28] Lastly, dispositional optimism has been associated with higher numbers of cytotoxic and helper T cells, higher lymphocyte count [29,30] and lower interleukin-6 response. When more optimistic individuals were given a typhoid vaccine, they exhibited greater antibody response.[31] No current studies have yet examined the interplay of obesity, stress, loneliness, social support, and dispositional optimism in response to the same vaccination.

Another consideration that may affect immune responses to vaccination include a person's STING variants. Animal studies have shown that Pneumovax23® efficacy is controlled by a gene called STING (stimulator of interferon genes)[41]. Human STING gene shows significant hetergenicity and population stratification[42]. Using animal models of human STING variants and human B cells from various human STING gene carriers, Dr. Jin's group discovered that human STING variants influence the efficacy of Pneumovax23®. These human STING genotypes account for ~10% of Caucasians, ~14% of Africans, ~18% of South Americans and ~30% of East Asians. These variants may therefore play a role in how participants' immune system respond to the vaccine.

4. RESEARCH PLAN & METHODS:

A. Experimental Design

1. Study Participants: For this study, we will recruit 86 participants. However, not all of those participants will qualify for the study and we estimate that, of the 86, a total of 56 people will qualify for the study. This accounts for a 20% attrition rate, reaching a desired 46 participants based on our power analysis. The 56 participants will be separated into two groups, each consisting of 28 participants. Group 1 will contain individuals with a BMI between 22 and 25. Group 2 will contain obese individuals, defined as a BMI $\geq$ 30 and waist to hip ratio of at least 0.9 in males and at least 0.85 in females. Study participants will be between the ages of 18-35 and in good health.

Recruitment Process: Participants will be recruited in the study under the title, "Pneumonia Vaccine Study."

Flier - Fliers will be posted on boards and walls at the University of Florida Health Science campus and all affiliated or satellite sites as well as in busy areas with lots of foot traffic within the community (Butler Plaza, Publix, Oaks Mall, etc.).

IDR - If we require more numbers, the IDR will be utilized to contact individuals who fit the criteria for our study. Our co-investigators will be calling each eligible person.

Inclusion Parameters: Alachua county resident

Said yes to recontact for research studies

Obesity diagnosis

Exclusion Parameters: Diabetes, complicated

Drug Abuse

Weight Loss

AIDS

Alcohol abuse

Renal failure

Solid tumor without metastasis

Metastatic cancer

Liver disease

Lymphoma

Leaves 150 possibly eligible participants to contact

ResearchMatch – ResearchMatch allows researchers to search for volunteers who have identified themselves as being interested in participating in research. Interested participants create a "profile"

with basic health information that allows researchers to search for potential participants that meet certain criteria. If a match is identified, the potential participant can be sent details about the study opportunity and can then choose whether to provide their contact information to the researcher. There is an estimated 30 potential participants to contact who are located within 25 miles of UF, between 18 and 35 years of age, have a BMI of 30+, do not smoke, and report no medical conditions.

Health Street – If we require more numbers, we will also recruit local community members through Health Street. There is an estimated 400 people who meet eligibility criteria (age range, BMI, non-diabetic). HealthStreet identifies potential participants in a database based on their health conditions reported at time of intake. HealthStreet contacts them, reads a brief description of the study with them over the phone, as well as general inclusion/exclusion criteria that are not recorded on their intake. The members are asked if they are interested in participating, and they are informed that the study coordinator will contact them for screening to determine if they are eligible or to schedule an initial appointment. HealthStreet sends the member's contact information to the coordinator using an encrypted spreadsheet. The coordinator then contacts the potential participant by phone within two weeks and informs HealthStreet whether the participant enrolled or was excluded.

Presenting – If we require more numbers, we will present our study to University of Florida students and staff in hopes of recruiting from that audience, pending permission to take up class time. The same powerpoint mentioned above may be utilized.

Craigslist - We will place ads on the Gainesville Craigslist website. If we require more numbers, ads will also be placed in the local newspapers, *The Alligator* and *The Gainesville Sun*.

UF PBS – If we require more numbers, we will run a 15 second public service announcement on the NPR public radio station WUFT-FM 89.1.

Interested individuals will be instructed to call the phone number (352-247-2437) or email PneumoniaVaccine-Study@ufl.edu to reach one of the co-investigators.

Screening: Eligibility will be determined by phone or through a questionnaire via RedCap (depending on participant preference). We will acquire weight and height information to calculate BMI, as well as ask if participants believe they are still eligible after listening to a list of our exclusion criteria.

At the end of the screening, we will request an email address in order to email a copy of the consent form for the participant to review before the first visit, during which the consent form will be reviewed with the participant and any questions addressed. We will also request a cell phone number at which we can text participant reminders the night before their appointments (visit 1 and visit 2). If the participant declines a reminder text, we will have a reminder phone call, if they decline this, we will send email reminders.

Consenting: Upon arriving the co-investigators will have a discussion with the participants about the study. Any questions or concerns will be appropriately addressed. All co-investigators obtaining consent will be trained on obtaining informed consent through the CTS800 CTSI Informed Consent Training. Dr. Brantly will provide supervision.

Confirmation of Screening Accuracy: During the first visit, a pregnancy test will be given to females and an HbA1C will also be taken via finger prick to assess blood sugar levels. If the participant is not pregnant and has an HbA1C less than 6.5%, then comprehensive physical exams will be performed and vitals will be taken to assess health. If the participant is found to be pregnant, to have an HbA1C greater than 6.5%, to have a positive physical exam or comprehensive past medical history finding the participant will be considered a “screening failure”. Vials from the blood draw will be sent to Shands for a CBC and CMP. If there is a clinically significant CBC or CMP finding as determined by Dr. Mark Brantly, then the participant will be considered a “screening failure.” CBC/CMP values are being considered because these values will impact the conclusions we can draw from the data. These values are not necessary to determine if there are any contraindications to the vaccine. If screening failure occurs, then the participant will ineligible to continue with the study based off of failure to meet inclusion criteria.

2. Study Design: Participants will be asked to fast after midnight prior to the first blood draw. A baseline blood draw (39 mL) will be performed for each participant. Pneumovax 23 will then be administered into the deltoid muscle by the CRC nurses; needle length will be adjusted according to distance from skin surface to muscle bundle. The Pnueumovax 23 vaccine will be provided by the University of Florida Investigational Drug Services. They receive their commercial products from McKesson Pharmaceuticals. Effort will be taken to esure that all participants receive the same lot number of Pneumovax 23.

Blood will be drawn 4-6 weeks following vaccination, as primary antibody production peaks during this time frame.[40] At that time, a total of 33 mL of blood will be drawn for analyses of antibody and cytokine levels. Table I illustrates what assays will be performed on the blood drawn at each time point and Table II illustrates the schedule of each blood draw. Figure 2 outlines a progression of events that will occur during both the screening and the first visit.

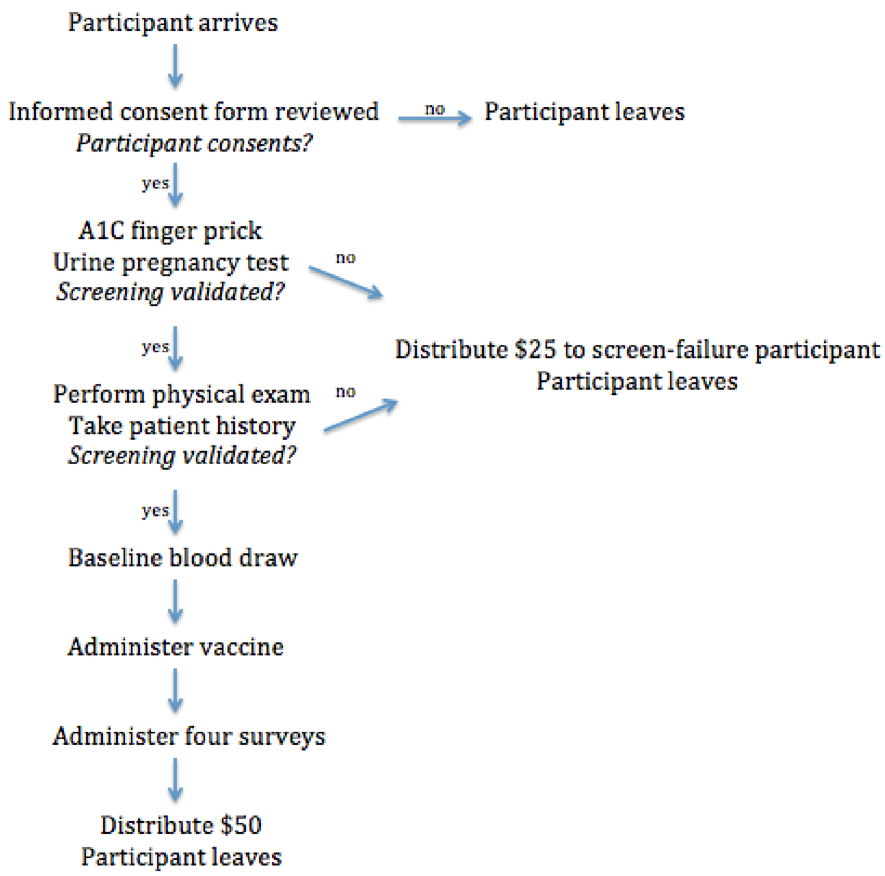

Figure 2. Progression of events during Visit 1:

Consenting, screening confirmation, blood draw,

vaccination, survey administration, and payment distribution

B. Measurements to be Collected

1. Basic Demographics (e.g. gender, age, race, ethnicity, education, etc.) will be collected.
2. Height, weight and hip-waist circumference will be collected.
3. Baseline Characteristic Assessments: Pregnancy will be evaluated by urine test, and hemoglobin A1C level will be determined with a portable finger-prick monitor.
4. Whole Blood Lab Assays: The following whole blood lab assays will be sent to and performed by Shands Laboratory: total IgG titers, complete metabolic panel, erythrocyte sedimentation rate, and complete blood count (CBC).
5. Anti-pneumococcal Antibodies: IgG antibody titers specific for each serotype in Pneumovax 23 (i.e. 1, 2, 3, 4, 5, 6B, 7F, 8, 9N, 9V, 10A, 11A, 12F, 14, 15B, 17F, 18C, 19F, 19A, 20, 22F, 23F, and 33F) will be analyzed using a panel ordered through the Mayo Clinic.
6. Serum Analyte Measurements: ELISA will be used to measure various acute phase reactants (e.g. TNF, C reactive protein, interferons, interleukins, etc.) and Ig subclasses. If significant

differences are not observed between arms then a cytometric bead assay may be employed. ELISAs will be performed in Dr. Mark Brantly's lab.

7. The Perceived Stress Scale-10, which is a 10-item scale assessing how stressful participants perceive their lives to be. Scores range from 0 to 40. This scale has shown adequate reliability (alpha = 0.78).[32]
8. The UCLA Loneliness Scale, a 20-item scale that measures subjective feelings of loneliness and social isolation. Scores range from 0 to 60. This scale has shown adequate reliability (alpha = 0.89 to 0.94) with one year test-retest reliability of r = 0.73.[33]
9. The Interpersonal Support Evaluation List-12, a 12-item scale that measures perceived social support along 3 dimensions: appraisal support (availability of someone to talk to), belonging support (availability of people to do things with), and tangible support (e.g. material aid). Scores range from 0 to 36. This scale has shown adequate reliability (alpha = 0.88 to 0.90 in the general population).[34]
10. The Life Orientation Test-Revised), a 10-item scale measuring optimism versus pessimism along a continuous scale.[35] Scores range from 0 to 40.
11. Genetic Sequencing: the human *STING* gene will be sequenced.

<u>Protocol for Collection and Storage of Venous Blood</u>

The blood volume will be distributed between Shands' Laboratory, the Mayo Clinic's Laboratory, and Dr. Brantly's Lab to assess CMP, CBC, antibody responses, and perform experiments.

Table 1. Itemized Details of Laboratory Specimen Collection

Test	Amount Needed	Collection	Test Location	Location of Draw	Time of Test
CMP	3 mL	Light-green tube	UF Health	CRC	Visit 1
CBC and ESR	3 mL	Purple tube	UF Health	CRC	Visit 1
Total Immunoglobulins	3 mL	Light-green tube	UF Health	CRC	Visit 1 & 2
Pneumovax Serotypes	5 mL	Gold Tube	Mayo Clinic	CRC	Visit 1 & 2
Cytokines and inflammatory markers (i.e. IL-6, IL-12, IL-4, , etc.)<br><br>Antibody titers: IgG (isotypes); IgG1, IgG2, IgG3, IgG4, and	15 mL	Gold Tube	Brantly Lab	CRC	Visit 1 & 2

IgM.					
Genetic testing to further assess immune response (ie STING genotyping analysis etc.) and future inflammatory analysis	10 mL	Purple Tube	Brantly Lab	CRC	Visit 1 & 2
**Total Blood Drawn**	**Visit 1: 39 mL**	**Visit 2: 33mL**			
A1C	finger-prick		CRC	CRC	Visit 1
Hcg Pregnancy Test	20-50 mL	Urine Collection Cup	CRC	CRC	Visit 1

C. Data Storage and Confidentiality

All data collected for enrolled participants will be deidentified and stored for at least ten years. Hard copies will be kept in binders that will be locked and secured in the laboratory of Dr. Mark Brantly. Electronic copies will stored in REDCap. Additionally, information pertinent to the participant's medical history will be documented in EPIC. Furthermore, all systems that will make use of any data from this project will be UF-approved devices that have sufficient anti-virus and encryption software installed. For enrolled participants who do not complete the study for any reason, data will also be stored for ten years, unless specifically requested by the participant to be destroyed. In this case, electronic data will be deleted, and hard copies will be shredded. If volunteers do not qualify for the study based on the initial screen, their information will be deleted and hard copies will be shredded. Otherwise stated, all data collected during the screening phase will be eliminated if the potential participant in question does not sign the informed consent document. Table 2 details where information will be stored at each step throughout the study.

Table 2. Data Storage Locations

	Patient Binder – Storage for 10 years	Redcap	EPIC
**Screening**			
Qualifying form	X	X	X
**Visit 1 & Screening Confirmation**			
Informed Consented	X	X	
A1C and Pregnancy Test	X	X	
Vitals	X	X	X

H&P	X		X
Payment Information	X	X	
Vaccination	X	X	X
**Visit 1 & 2**			
Laboratory and Results			
Shands	X	X	X
Mayo	X	X	
Brantly	X	X	
Surveys	X	X	
Adverse Events	X	X	

D. Setting and Facilities

Participants will be asked to report to the outpatient rooms of the University of Florida's Clinical and Translational Research Building (CTRB) located at 2004 Mowry Rd, Gainesville, FL 32610. At the CRC participants will be consented and vitals, biological samples, and surveys will be collected.

E. Limitations of the Study

Since Pneumovax 23 is normally recommended for individuals of age ≥ 50 or > 2 years who are at increased risk for pneumococcal disease and approved for use in individuals age 50 and older, there may be intrinsic differences in the immune system's response to the vaccine as opposed to our study population (aged 18-35). However, the present study is a necessary pilot before examining the effect of obesity on Pneumovax 23 vaccination in the elderly population, which will necessarily be more complex due to the multitude of covariates that will need to be studied and/or controlled for.

F. Limitations of Study Design

One primary limitation in the study design is the inability to draw conclusions about causation. Given that there is no randomization in the study design, we can only draw conclusions about association via correlation. That is, we can only conclude that obesity is associated with an altered immune response, not that it causes it.

Another limitation is that we will likely be unable to examine the exact point at which various immune-marker levels peak. With 2 blood draws separated by 4-6 weeks, we can only approximate this time point. However, our primary outcome measure is relative differences in immune-marker levels between the normal and obese group. For this calculation, peak levels and their associated time point are not crucial.

G. Safety Procedures, Risk/Benefit Assessment, Payment

Receiving the Pneumovax 23 vaccine is beneficial in case a participant is exposed to *Streptococcus pneumoniae* bacteria at any point after the study.

Others may benefit from the study in that elderly patients and immuno-compromised patients are more susceptible to infection with pneumonia and are generally more resistant to the benefits of vaccination. As the rate of obesity increases, the proportion of elderly obese will also be increasing. This leads to a higher population at increased risk of contracting pneumonia. This data generated from healthy patients could be used to increase our understanding of the effect of obesity on immune function and ultimately advise physicians on how body weight may impact immune response to the Pneumovax 23 vaccination.

Possible risks include an allergic reaction at the Pneumovax 23 injection site, an air embolism, injection site swelling or bruising, fainting from blood draws, and infection at the injection site.[36] The most common side effects of the Pneumovax 23 vaccine itself are: pain, warmth, soreness, redness, swelling, hardening at the injection site, headache, weakness, feeling tired and muscle pain.[3] Should they occur, all of the co-investigators will review these adverse events with the PI once a week during the duration of the study, in accordance with our data safety monitoring plan. Adverse events will be logged in the regulatory binder. While the CBC/CMP test results are used to control for factors that might impact the questions the study is asking, any concerning CBC/CMP values will be discussed with the Dr. Brantly and the appropriate measures will be taken such as strongly encouraging participants to see their physician.

In addition to the effects that could potentially ensue following vaccination, participants may experience some acute psychological stress when recalling personal experiences to answer the questionnaires. Since these questionnaires are not clinical questionnaires designed to diagnose participants, there is no clear cut-off between normal and abnormal values. However, if someone were to score incredibly high on stress and loneliness and incredibly low on social support, Dr. Brantly will be consulted on whether it is an emergency situation and appropriate measures will be taken, such as referral for counseling.

Participants will be reimbursed up to $100. Installments will be given at the end of each visit.

Participants that are found to be ineligible due to exclusion criteria will receive $25 at the first visit. Otherwise, participants that meet all inclusion and exclusion criteria will receive $50 at the end of the first visit. All participants that return to the second visit will receive an additional $50. Participants receiving payment will have their social security numbers collected for financial purposes.

H. Subject Costs

There are no anticipated costs to the participants other than the time needed to participate in the study.

I. Consent Form

See attached informed consent form.

Exclusion Criteria:

The following criteria are used to exclude potential participants:

1. Pregnancy
    a. Pregnant
    b. Planning to become pregnant
    c. Breastfeeding
2. Diabetes mellitus (A1C> 6.5%)
    a. Diagnosed with diabetes
3. Fasting
    a. Inability to fast for 8 hours prior to the initial blood draw
4. Immunization
    a. Immunized with Pneumovax 23
    b. Immunized with Prevnar
5. Medications
    a. Currently taking any of the following medications or within the 4 weeks of study:
        i. Immunosuppressive drugs or chemotherapeutic agents including azathioprine, mycophenolate mofetil, TNF inhibitors, cyclosporine, methotrexate, leflunomide, cyclophosphamide, sulfasalazine, antimalarial drugs, chlorambucil, and nitrogen mustard.
        ii. Steroids including prednisone, cortisone, and hydrocortisone.
6. A history of clinical illness, as determined by a comprehensive patient history at the first visit.
    a. Pneumonia
        i. Previously had Pneumonia
    b. Low blood pressure
        i. Experienced dizzy spells
    c. Significant cardiovascular disease
        i. Recently hospitalizations deemed significant at the discretion of the Principal Investigator
        ii. History of heart attack
        iii. History of stroke
    d. Spleen
        i. Splenectomy
        ii. Damage to spleen
    e. Lung Disease
        i. History of lung disease
    f. Kidney disease
        i. Blood in urine
        ii. Protein in urine
    g. Liver disease
        i. Previous or current liver diseases including hepatitis A, B, or C, liver cirrhosis, or autoimmune hepatitis
    h. Leukopenias
    i. Hemoglobinopathies
        i. History of sickle cell disease, thalassemia
    j. HIV/AIDS
        i. History of HIV infection

k. Recreational IV drug use
    i. IV drug use is a risk factor for HIV infection, which may change the immune response to Pneumovax23.
l. History of idiopathic thrombocytopenic purpura
m. History of chronic inflammatory diseases including rheumatoid arthritis or chronic granulomatous disease
n. Autoimmune disease
o. Immunodeficiencies
    i. e.g. neutropenia, common variable immune deficiency, Bruton's X-linked agammaglobulinemia; SCID; Selective IgA deficiency; Wiskott-Aldrich Syndrome; Ataxia Telangiectasia; DiGeorge Syndrome; Chronic Granulomatous Disease; Hyper IgE Syndrome; Complement deficiencies

7. Weight
    a. Planning on losing weight within the time frame of the study
    b. Have experienced excessive weight loss or gain within two months prior to the study
    c. Planning to undergo liposuction, gastric bypass, stomach stapling, whipple procedure, or reconstructive surgery involving transplantation of adipose tissue within the study period
    d. Bodybuilder or someone who takes part in excessive weight training
8. Allergy
    a. Any severe side effects from vaccines
    b. Allergic reaction to phenol
9. Medications/Supplementations
    a. Taking any antioxidant supplements (EmergenC)
    b. Unable to refrain from antioxidant supplements throughout the duration of the study

A physician, Dr. Mark Brantly, will be immediately notified in the event of anaphylaxis or severe changes in vitals (blood pressure above 180/110 or below 80/50 mmHg, pulse above 120 or below 45 per minute, temperature above 101F or below 94F, or respiration above 30 or below 8 per minute).

Inclusion Criteria:

The control group will consist of individuals aged 18-35 with BMIs between 22-25kg/m^2, as this BMI range was shown as optimally healthy and inversely related to overall mortality risk in a meta-analysis of over 900,000 individuals.[37] The obese group will consist of individuals with BMIs greater than 30kg/m^2 and waist to hip ratio of at least 0.9 in males and at least 0.85 in females, in accordance with the CDC and WHO guidelines for defining obesity.[38,39] The participants will be subjected to one vaccination and two blood draws over the course of the study. Additionally, they will complete a total of four surveys to assess perceived stress, loneliness, social support, and optimism.

5. STATISTICAL DESIGN AND CONSIDERATIONS

A. Design

The study will include a control and experimental group. Briefly, the control group consists of healthy, normal-weight individuals and the experimental group consists of obese individuals. After passing an

initial screening, eligible participants will be asked to come in for the first of two visits. During the first visit, informed consent will be reviewed, and the informed consent form will be signed. Subsequently, vital signs, patient history, blood, and other baseline measures will be taken. If the urine pregnancy test and A1C test (< 6.5%) confirm the initial screen results, and if the physical exam and history findings are satisfactory, participants will receive a single dose of Pneumovax 23. The participants will then return for one additional blood draw 4-6 weeks after the first visit. The participants will be physically present for a total of 2 visits and will experience 2 total blood draws. If at any time participants become pregnant, diabetic, or are found to have any conditions listed in the exclusion criteria as revealed by CBC, CMP, or physical exam, then subjects will be indicated as screening failures and will not be included in data analysis due to failure to meet inclusion criteria.

B. Hypotheses

Primary Hypothesis (stated as null): Levels of antibodies to serotypes in Pneumovax 23, in pre- and post- vaccination in healthy BMI individuals and obese individuals are identical.

Secondary Hypothesis (stated as null): Levels of pro- and anti-inflammatory cytokine levels pre- and post- vaccination in healthy weight obese and groups are identical.

Tertiary Hypothesis (stated as null): The perceived level of stress, loneliness and social support will be the same between the healthy weight and obese groups or between groups with different immune responses to the vaccine.

Quaternary Hypothesis (stated as null): Those who carry STING variants and those who are wildtype STING carriers will not have differing immune responses to Pneumovax 23.

C. Statistical Methods

To test the primary hypothesis, we will use the Mayo criteria for positivity of 23 titers, with the primary outcome being the number of positives. We shall employ a two-sided ANCOVA, adjusting for age and gender to compare the obese cases to the non-obese controls. Secondarily, we will compare the 23 individual quantitative titers by similar ANCOVA analyses. Other endpoints will be analyzed in a similar manner as tertiary outcomes.

D. Randomization

No randomization will be done in this study.

E. Sample Size

We expect that 23 evaluable obese participants and 23 evaluable normal weight participants will complete the study. To accommodate for 20% attrition rate we will recruit 28 obese participants and 28 normal participants for a total of 56 eligible subjects. Additionally, to accommodate for individuals unable to participate due to exclusion criteria or individuals eliminated post-enrollment due to screening failure (e.g., CBC/CMP abnormalities), we plan to screen an additional 30 subjects. This accounts for a total of 86 individuals screened. Using information from the 2013-2014 MD-PhD study, we compared those with BMI <25 vs. those with BMI > 25. The distribution of Positive titers amongst the 14 was as follows:

BMI	5	6	7	8	9	10	11	12	Total
<25	1	2	0	2	2	1	4	2	14
>25	0	2	3	0	1	1	0	0	7

This should be viewed as conservative, as we will have greater separation of BMI values than the prior study. The actual gain in power cannot be quantified.

F. Technical Support

Under a CTSI voucher, Dr. Jonathan Shuster assisted with the statistics and data analysis. Dr. Shuster co-authored the statistical section with the students. We will use CTRB facilities for blood draw, temporary tissue storage, and basic lab protocols. The laboratory of Dr. Mark Brantly, MD, will be used to perform experimental procedures including the ELISA assays for IgG quantification.

6. REQUIRED REPORTING

The following people will be responsible for all protocol amendments, updated consent forms and IRB approval letters; electronic updates of protocols, reporting data safety monitoring activities: Leanne Dumeny, Robert Eisinger, Hunter Futch, Chu Jane Hsiao, and Mathew Sebastian.

7. TIME AND EVENTS

Potential participants will be screened via phone or REDCap surveys. If a potential participant qualifies after the screening survey, a visit to the UF campus CRC building will be scheduled.

Upon arrival at the CRC, informed consent will be reviewed, and the informed consent document will be signed. Following this enrollment, the participant will have vitals checked and will undergo an HbA1C fingerprick test, a urine pregnancy test, a physical exam, and a comprehensive medical history. If the participant still meets inclusion criteria, then screening accuracy is confirmed. Participants who are found to be screening failures will be told they are no longer eligible for the second part of the study and be given a gift card. For those that continue, blood will be drawn and sent to respective locations outlined in Table 1. Subsequently, the Pneumovax 23 vaccine will be administered. Following administration of the vaccine, four questionnaires will be administered; simultaneously while completing the forms, the

participant will be monitored for adverse side effects to the vaccine. Payment will be administered after the surveys, and the subsequent visit will be scheduled.

At the second visit 4-6 weeks following the first, blood will be drawn once more and distributed to the locations outlined in Table 1. After the blood draw, the participant will be compensated and thanked profusely for participating in the study. Completion of visit two marks the culmination of the study for that particular participant. Data analysis will ensue once 56 participants have progressed through the study. The events occurring in the study are outlined in Table 3.

Table 3. Proposed Schedule

	**Events**	**Prior to Visit 1**	**Estimated Time (min)**	**Visit 1: Day 0**	**Visit 2: Wk 4-6**
**Screening**	Contact with participants by phone or email	X	10		
	Set up REDCAP and chart for each participant	X	20		
	Screening via phone or REDCAP survey	X	10		
	Instructions email or phone call	X	10		
**Visit 1**	Informed Consent		20	X	
	Urine Pregnancy Test		10	X	
	A1C		5	X	
	Medical History		10	X	
	Physical Exam,		10	X	
	Waist/Hip Ratio		2	X	X
	Vitals		2	X	X
	Blood draw for CMP, CBC, and ESR		6	X	
	Pneumvax23 Vaccination		5	X	
	Blood draw for total IgG, serotypes, and cytokine assays		6	X	X
	Questionnaire Completion (4 surveys)		30	X	
	Instructions for next visit		5	X	
	Payment		5	X	X
	**Total Time Per Visit (min)**			114	20-30

8. CITATIONS

1. Zar, H. J., S. A. Madhi, S. J. Aston, and S. B. Gordon. (2013). "Pneumonia in low and middle income countries: progress and challenges." *Thorax* .

2. World Health Organizations (WHO). (2014).“Pneumonia: Fact sheet.” Geneva (Switzerland): *World Health Organization (WHO).*

3. Merck & Co., Inc. (1986). “Highlights of Prescribing Information. Pneumovax23.” *Merck & Co., Inc*.

4. Butler, J. C., R. F. Breiman, J. F. Campbell, H. B. Lipman, C. V. Broome, and R. R. Facklam. (1993). "Pneumococcal Polysaccharide Vaccine Efficacy: An Evaluation of Current Recommendations." *JAMA: The Journal of the American Medical Association* 270.15: 1826-831.

5. Hirschtick, R.E., Glassroth J., Jordan, M.C., Wilcosky, T.C., Wallace, J.M., Kvale, P.A., Markowitz, N., Rosen, M.J., Mangura, B.T., Hopewell, P.C. (1995) “Bacterial pneumonia in persons infected with the human immunodeficiency virus.” *The New England Journal of Medicine* 333: 845-851.

6. Baik, I., Curhan, G.C., Rimm, E.B., Bendich, A., Willett, W.C., Fawzi, W.W. (2000). “A Prospective Study of Age and Lifestyle Factors in Relation to Community-Acquired Pneumonia in US Men and Women.” *Archives of Internal Medicine* 160: 3082-3088.

7. Corey. K. E., and L.M. Kaplan. (2014). “Obesity and liver disease: the epidemic of the twenty-first century.” *Clinical Liver disease*. 10.1016/j.cld.

8. Hollinger, F.B. (1989). “Factors influencing the immune response to hepatitis B vaccine, booster dose guidelines, and vaccine protocol recommendations.” *The American Journal of Medicine* 87.3.S1: S36-S40.

9. Elaikim, A., Swindt, C., Zaldivar, F., Casali, P., Cooper, D.M. (2006). “Reduced tetanus antibody titers in overweight children.” *Autoimmunity* 39.2: 137-141.

10. Sheridan, P.A., Paich, H.A., Handy, J., Karlsson, E.A., Hudgens, M.G., Sammon, A.B., Holland, L.A., Weir, S., Noah, T.L., Beck, M.A. (2012). “Obesity is associated with impaired immune response to influenza vaccination in humans.” *International Journal of Obesity* 36:1072-1077.

11. Young, K.M., Gray, C.M., Bekker, L.G. (2013). “Obesity A Risk Factor for Vaccine Non-Responsiveness?” *PLoS ONE* 8.12: e82779.

12. Samartin, S., Chandra, R.K. (2001). “Obesity, overnutrition and the immune system.” *Nutrition Research* 21.1-2: 243-262.

13. Després, Jean-Pierre, and Isabelle Lemieux. (2006). "Abdominal obesity and metabolic syndrome." *Nature,* 444.7121: 881-887.

14. Lemieux, Isabelle, et al. (2001). "Elevated C-reactive protein another component of the atherothrombotic profile of abdominal obesity." *Arteriosclerosis, Thrombosis, and Vascular Biology* 21.6 : 961-967.

15. Singh, Gurmukh. (2014). "C-reactive protein and erythrocyte sedimentation rate: Continuing role for erythrocyte sedimentation rate." *Advances in Biological Chemistry 2014*

16. Hur, S. J., Kim, D. H., Chun, S. C., & Lee, S. K. (2013). "Effect of adenovirus and influenza virus infection on obesity." *Life sciences*, *93*(16), 531-535.

17.Karlsson, E. A., and M. A. Beck. (2010). "The Burden of Obesity on Infectious Disease." *Experimental Biology and Medicine* 235.12: 1412-424.

18. Talbot, H.k., L.a. Coleman, K. Crimin, Y. Zhu, M.t. Rock, J. Meece, D.k. Shay, E.a. Belongia, and M.r. Griffin. (2012). "Association between Obesity and Vulnerability and Serologic Response to Influenza Vaccination in Older Adults." *Vaccine* 30.26: 3937-943.

19. Glaser, R., and Kiecolt-Glaser, J. K. (2005). "Stress-induced immune dysfunction: implications for health." *Nature Reviews Immunology*, *5*(3), 243-251.

20. Padgett, D. A., & Glaser, R. (2003). "How stress influences the immune response." *Trends in Immunology*, *24*(8), 444-448.

21. Glaser, R., Kiecolt-Glaser, J. K., Bonneau, R. H., Malarkey, W., Kennedy, S., & Hughes, J. (1992). "Stress-induced modulation of the immune response to recombinant hepatitis B vaccine." *Psychosomatic Medicine*, *54*(1), 22-29.

22. Glaser, R., Sheridan, J., Malarkey, W. B., MacCallum, R. C., & Kiecolt-Glaser, J. K. (2000). "Chronic stress modulates the immune response to a pneumococcal pneumonia vaccine." *Psychosomatic medicine*, *62*(6), 804-807.

23. Kiecolt-Glaser, J. K., Glaser, R., Gravenstein, S., Malarkey, W. B., & Sheridan, J. (1996). "Chronic stress alters the immune response to influenza virus vaccine in older adults." *Proceedings of the National Academy of Sciences*, *93*(7), 3043-3047.

24. Hawkley, L. C., & Cacioppo, J. T. (2010). "Loneliness matters: a theoretical and empirical review of consequences and mechanisms." *Annals of Behavioral Medicine*, *40*(2), 218-227.

25. Pressman, S. D., Cohen, S., Miller, G. E., Barkin, A., Rabin, B. S., & Treanor, J. J. (2005). "Loneliness, social network size, and immune response to influenza vaccination in college freshmen." *Health Psychology*, *24*(3), 297.

26.Costanzo, E. S., Lutgendorf, S. K., Sood, A. K., Anderson, B., Sorosky, J., & Lubaroff, D. M. (2005). "Psychosocial factors and interleukin-6 among women with advanced ovarian cancer." *Cancer*, *104*(2), 305-313.

27. Friedman, E. M., Hayney, M. S., Love, G. D., Urry, H. L., Rosenkranz, M. A., Davidson, R. J., ... & Ryff, C. D. (2005). "Social relationships, sleep quality, and interleukin-6 in aging women." *Proceedings of the National Academy of Sciences of the United States of America*, *102*(51), 18757-18762.

28. Lutgendorf, S. K., Anderson, B., Sorosky, J. I., Buller, R. E., & Lubaroff, D. M. (2000). "Interleukin-6 and use of social support in gynecologic cancer patients." *International Journal of Behavioral Medicine*, *7*(2), 127-142.

29. Segerstrom, S.C., Taylor, S.E., Kemeny, M.E., and Fahey, J.L. (1998). "Optimism is associated with mood, coping and immune changes in response to stress." *Journal of Personality and Social Psychology*, 74(6), 1646-1655.

30. Cohen, F., Kearney, K. A., Kemeny, M. E., & Zegans, L. S. (1989). "Acute stress, chronic stress, and immunity, and the role of optimism as a moderator." In *Psychosomatic Medicine*, Vol. 51, No. 2, pp. 255-255.

31. Brydon, L., Walker, C., Wawrzyniak, A.J., Chart, H., and Steptoe, A. (2009). "Dispositional optimism and stress-induced changes in immunity and negative mood." *Brain, Behavior and Immunity,* 23(6), 810-816.

32. Cohen, S., Kamarck, T., and Mermelstein, R. (1983). "A global measure of perceived stress." *Journal of Health and Social Behavior*, 24:385-396.

33. Russell, D. (1996). "UCLA loneliness scale (version 3): Reliability, validity, and factor structure." *Journal of Personality Assessment*, 66: 20-40.

34. Cohen, S., Mamelstein, R., Kamarck, T., and Hoberman, H. (1985). "Measuring the functional components of social support." In I.G. Sarason & Sarason (Eds.), *Social support: Theory, research and application*.73-94.

35. Scheier, M. F., Carver, C. S., & Bridges, M. W. (1994). "Distinguishing optimism from neuroticism (and trait anxiety, self-mastery, and self-esteem): a reevaluation of the Life Orientation Test." *Journal of personality and social psychology*, *67*(6), 1063.

36. World Health Organization (WHO). (2010). "WHO guidelines on drawing blood: best practices in phlebotomy." Geneva (Switzerland): *World Health Organization (WHO)*; 109 p.

37. Prospective Studies Collaboration. (2009). "Body-Mass Index and Cause-Specific Mortality in 900 000 Adults: Collaborative Analyses of 57 Prospective Studies." *Lancet* 373.9669: 1083–1096.

38. Centers for Disease Control and Prevention (CDC). (2014). "Obesity and Overweight." *Centers for Disease Control and Prevention*.

39. World Health Organization. (2008). "Waist Circumference and Waist–Hip Ratio: Report of a WHO Expert Consultation." World Health Organization, 39p.

40. Siegrist, C.A. (2008) "Vaccine immunology." In *Vaccines*, ed. S Plotkin, W Orenstein, P Offit, pp. 17–36. Philadelphia, PA: Saunders Elsevier. 5th ed.

41. Zeng M, et al. (2014) MAVS, cGAS, and endogenous retroviruses in T-independent B cell responses. Science 346(6216):1486-1492.

42. Jin L, et al. (2011) Identification and characterization of a loss-of-function human MPYS variant. Genes and immunity 12(4):263-269.
